# Low Proviral Load is Associated with Indeterminate Western Blot Patterns in Human T-Cell Lymphotropic Virus Type 1 Infected Individuals: Could Punctual Mutations be Related?

**DOI:** 10.3390/v7112897

**Published:** 2015-10-28

**Authors:** Camila Cánepa, Jimena Salido, Matías Ruggieri, Sindy Fraile, Gabriela Pataccini, Carolina Berini, Mirna Biglione

**Affiliations:** Instituto de Investigaciones Biomédicas en Retrovirus y SIDA, UBA-CONICET, Paraguay 2155, piso 11, C1121ABG, CABA, Argentina; canepa.camila@gmail.com (C.C.); jimenasalido@gmail.com (J.S.); ruggierimatias50@gmail.com (M.R.); sfraile77@gmail.com (S.F.); gaby.pataccini@hotmail.com (G.P.); cberini@fmed.uba.ar (C.B.); mbiglione@fmed.uba.ar (M.B.)

**Keywords:** HTLV-1/2, proviral load, Western blot, indeterminate, mutations

## Abstract

Background: indeterminate Western blot (WB) patterns are a major concern for diagnosis of human T-cell lymphotropic virus type 1 (HTLV-1) infection, even in non-endemic areas. Objectives: (a) to define the prevalence of indeterminate WB among different populations from Argentina; (b) to evaluate if low proviral load (PVL) is associated with indeterminate WB profiles; and (c) to describe mutations in LTR and *tax* sequence of these cases. Results: Among 2031 samples, 294 were reactive by screening. Of them, 48 (16.3%) were WB indeterminate and of those 15 (31.3%) were PCR+. Quantitative real-time PCR (qPCR) was performed to 52 HTLV-1+ samples, classified as Group 1 (G1): 25 WB+ samples from individuals with pathologies; Group 2 (G2): 18 WB+ samples from asymptomatic carriers (AC); and Group 3 (G3): 9 seroindeterminate samples from AC. Median PVL was 4.78, 2.38, and 0.15 HTLV-1 copies/100 PBMCs, respectively; a significant difference (*p*=0.003) was observed. Age and sex were associated with PVL in G1 and G2, respectively. Mutations in the distal and central regions of Tax Responsive Elements (TRE) 1 and 2 of G3 were observed, though not associated with PVL.The 8403A>G mutation of the distal region, previously related to high PVL, was absent in G3 but present in 50% of WB+ samples (*p* = 0.03). Conclusions: indeterminate WB results confirmed later as HTLV-1 positive may be associated with low PVL levels. Mutations in LTR and *tax* are described;  their functional relevance remains to be determined.

## 1. Introduction

Human T-cell lymphotropicvirus type 1 and 2 (HTLV-1/2) are distributed worldwide. HTLV-1 infects an estimated 20 million people in the world and is considered the etiologic agent of adult T-cell leukemia/lymphoma (ATLL), HTLV-associated myelopathy/tropical spastic paraparesis (HAM/TSP), and HTLV-1 uveitis [1]. HTLV-1 presents foci of endemicity in the Caribbean, Southeastern Japan, sub-Saharan Africa, the Middle East, and areas of South America, while HTLV-2 is naturally endemic in natives from Africa and aborigines of the Americas [1,2]. Concerning phylogeny, seven subtypes have been identified within HTLV-1: cosmopolitan (a), Central African (b and d), Melanesian (c), a variant from Zaire (e), one from Gabon (f), and one from Cameroon (g). The cosmopolitan subtype, disseminated worldwide, is composed of five subgroups: transcontinental (A), Japanese (B), West African (C), North African (D), and Black Peruvian (E) [3,4,5]. In Argentina, HTLV-1 cosmopolitan subtype transcontinental subgroup A is the major subgroup detected in the endemic area of thenorthwest as well as in blood donors, pregnant women, and different at-risk populations in non-endemic regions [6,7].

Mandatory screening for HTLV-1/2 in blood banks, which includes detection by an enzyme immunoassay (EIA) or particle agglutination (PA), has been implemented in many countries so far**.** According to the current algorithm, a serological confirmation, usually by Western blot (WB), should be performed after reactive screening results [8]. However, despite improvements made in the WB assay specificity over the past years, HTLV-indeterminate WB results continue to be frequent in blood donors, mainly in inter-tropical areas, posing a major challenge for routine diagnosis worldwide [9,10,11]. 

It has been observed that the use of screening tests with low specificity significantly increases the number of indeterminate WB results that are later confirmed negative for the infection by molecular techniques [12]. Other possible explanations include cross-reactivity against other retroviruses or microbial agents, as occurs with *Plasmodium falciparum* in Central Africa, Indonesia, and the Philippines [13,14,15]. Regarding seroindeterminate cases later confirmed positive for the infection, several hypotheses have been proposed such as the presence of defective virus or low copy numbers of prototypic HTLV-1/2 that could be yielding a light antibody response [16]. Punctual mutations in key viral genes could be another alternative. Netto *et al*. have reported an association between G232A in the Tax Responsive Element (TRE) 1 and an increase in PVL levels [17]. Furthermore, it has been observed that non-synonymous mutations of the HTLV-1 *tax* gene could display markedly attenuated abilities to transactivate the provirus [18].

Over the last decade, a sensitive and specific nested polymerase chain reaction (n-PCR) assay able to confirm HTLV-1/2 infections in individuals with an indeterminate profile or HTLV positive but not typeable results by WB became an important tool for diagnosis [19,20]. Years later, quantitation of HTLV-1 proviral load (PVL) by quantitative real-time PCR (qPCR) was implemented for the follow-up of patients with associated pathologies worldwide [21,22,23]. Recently, both qPCR and multiplex (mqPCR) have been proposed as molecular testing for the confirmation of HTLV-1/2 diagnosis, aimed to address the issue of indeterminate results. However, according to reported data, these techniques still show sensibility problems [24,25].

As a consequence of frequent indeterminate WB results leading to difficulties in interpretation and counseling in our country, this study aims to (i) define the prevalence and banding profile frequency of cases with indeterminate results by WB among different populations from Argentina; (ii) evaluate whether a low PVL in HTLV-1 positive individuals is one of the causes of these results; and (iii) identify the presence of punctual mutations, both in Long Terminal Repeats (LTR) and *tax* regions, of indeterminate cases.

## 2. Results

### 2.1. Prevalence Studies

Prevalence of WB indeterminate results corresponding to samples from four different populations of Argentina is shown in Table 1. Three of them, Men who have Sex with Men (MSM), Injecting Drug Users (IDUs), and Female Sex Workers (FSW), belong to a previous epidemiological study [26]. The remaining one, the HTLV Diagnosis and Confirmation population (HDC), is composed of individuals referred from blood banks or hospitals to our Institute. The global methodology, including the number of samples tested at each step, is illustrated in Figure S1. The total number of WB indeterminate samples (IS) and, of those, the ones that were later confirmed as HTLV-1 or HTLV-2 positive by molecular techniques, are also shown in Table 1. 

**Table 1:** Prevalence of HTLV-1/2 infection and frequency of WB indeterminate patterns in four populations of Argentina. MSM: Men who have Sex with Men; IDU: Injecting Drug Users; FSW: Female Sex Workers; HDC: samples received at a Reference Institute for HTLV Diagnosis and Confirmation (HDC) from blood banks or hospitals of Argentina. ELISA: enzyme-linked immunosorbent assay. PA: particle agglutination.

	Reactive by PA or ELISA	Indeterminate samples (IS) n (%)	IS Confirmed HTLV-1+ by n-PCR n (%)	IS Confirmed HTLV-2+ by n-PCR n (%)	Total HTLV-1 Prevalence % (n/N)	Total HTLV-2 Prevalence % (n/N)
MSM (*N*=667)	26	11 (1.65)	3 (27.28)	0 (0)	0.45% (3/667) ^c^	0% (0/667)^c^
IDU (*N*=173)	36	4 (2.31 )	4 (100 )	2 ^b^ (100 )	4.62% (8/173) ^c^	15.6% (27/173) ^c^
FSW (*N*=613)	25	3 (2.12)	3 (23.10 )	0 (0 )	1.46% (9/613) ^c^	0.2% (1/613) ^c^
HDC (*N*=578)	207	30 (5.19 )	3 (15.79) ^a^	2 (10.53)^a^	18.8% (109/578)	5.36% (31/578)
Total	294	48 (16.33)	13 (35.13)	4 (10.81)	6.35% (129/2031)	2.90% (59/2031)

^a^Out of 30 seroindeterminate samples, only 19 could be tested by molecular techniques, as no DNA was available for the other 11. ^b^These two samples were HTLV-1/2 co-infected. ^c^Data reported by Berini *et al*. 2007 [26].

The different WB indeterminate banding patterns are detailed in Table 2 for all samples from the HDC population (*n*=30), including positive and negative ones by n-PCR. The banding patterns corresponding to the other three populations were previously described by Berini *et al*. 2007 [26].

**Table 2:** Description of WB indeterminate patterns for positive and negative samples by n-PCR among 578 samples received at a Reference Institute for HTLV Diagnosis and Confirmation (HDC) from blood banks or hospitals of Argentina.

WB Indeterminate Banding Pattern	N	HTLV-1/2 Negative	HTLV-1/2 Positive	Not Performed
GD21	6	3	1	2
GD21 + others	7	3	3	1
rgp46-1 and/or 2	4	1	1	2
p19	2	2	0	0
p19 + p24	4	2	0	2
p19 + others	1	0	0	1
HGIP	6	3	0	3
Total	30	14	5	11

**Figure 1:**
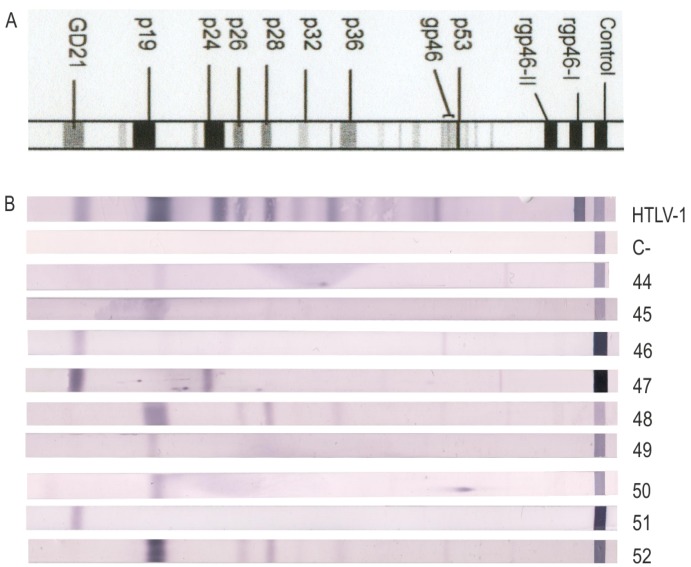
Western blot patterns of indeterminate cases confirmed HTLV-1 positive by n-PCR. Seroreactivity pattern using the MPD HTLV Blot 2.4 kit, which contains a recombinant GD21 (common for HTLV-1 and HTLV-2) and two synthetic peptides (rpg46-I and rpg46-II), specific either for HTLV-1 or HTLV-2. “HTLV-1”: HTLV-1 positive control. “C-“: negative control. 44–52: banding profile for each of the nine seroindeterminate cases analyzed.

The following experiments were performed in nine out of 13 seroindeterminate confirmed HTLV-1 positive cases found among the four studied populations, as samples from the other four were scarce. Figure 1 shows the banding pattern in each case.

### 2.2. Performance of the qPCR

The qPCR quantitation limit was 34 albumin copies/reaction and three *pol* copies/reaction. Samples with seroindeterminate results were run together, and an additional dilution was added to *pol* standard curve, as lower Threshold Cycle (Ct) values were expected. Acceptance criteria were accomplished and linearity was maintained (R^2^> 0.99). The intra-assay coefficient of variation (CV) was directly proportional to viral load levels: 14% at high load (>10 HTLV-1 copies/100 cells), 9% at medium load (1–10 HTLV-1 copies/100 cells), and 7% at low load (<1 HTLV-1 copy/100 cells); inter-assay CV at high loads was 24%. Of the 52 samples, PVL was detected in 51 and successfully quantified in 44 of them, including 25 pathology cases that were not in treatment at the time of the sample extraction (G1), 15 samples from asymptomatic carriers (AC) (G2), and four from seroindeterminate cases (G3), also AC. PVL was detected but not quantified in three G2 and four G3 samples, as the acceptance criteria was not met because of variable Ct values for *pol* gene. This viral gene was not detected in one of the G3 samples, although it could be amplified by n-PCR.

### 2.3. PVL Values

**Figure 2:**
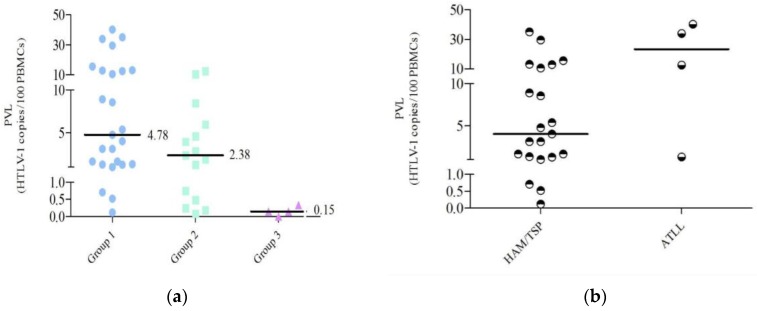
Individual proviral load values (PVLs). PVLs are expressed as HTLV-1 copies/100 PBMCs. (**a**) Samples are classified as Group 1: positive samples by Western blot (WB) from individuals with pathology that are not on treatment (*n* = 25), Group 2: positive samples by WB from asymptomatic carriers (AC) (*n* = 15), and Group 3: indeterminate samples by WB from AC (*n* = 4); PVL values are shown. A significant difference is observed between the three groups (p= 0.003) (GraphPad Prism V5). (**b**) PVLs for samples of Group 1, classified by disease: ATLL (*n* = 4) or HAM/TSP (*n* = 21) are shown, p= 0.07.

Individual PVL values are shown in Table 3. Median PVL values and standard errors were 4.78 (2.4), 2.38 (1.02), and 0.15 (0.07) HTLV-1 copies/100 PBMCs for G1, G2, and G3, respectively; a significant difference could be observed between the three groups (*p* = 0.003), as shown in Figure 2a. The difference was also significant (*p* = 0.005) when samples with seropositive WB results were analyzed as a single group [G1 + G2]. As shown in Figure 2b, no significant difference (*p* = 0.07) was observed when samples from acute leukemia (ATL; *n* = 4) were compared with HAM/TSP ones (*n* = 21).

### 2.4. PVL Distribution

Regarding WB indeterminate samples, all successfully detected PVLs (8/9) were lower than 1 HTLV-1 copy/100 cells. Concerning G1 and G2 samples, an overlap in the range of PVL values was observed (Table 4). In three cases, viral loads below 1 HTLV-1 copy/100 cells were observed in individuals with pathology (12%).

**Table 3:** Age, gender, and individual proviral load values (PVL) of cases confirmed as HTLV-1 positive by nested PCR (n-PCR). Samples were classified as Group 1: positive samples by WB from individuals with pathology that are not on treatment (*n* = 25), Group 2: positive samples by WB from asymptomatic carriers (AC) (*n* = 18), and Group 3: indeterminate samples by WB from AC (*n* = 9). Codes for 14 LTR and/or *tax* sequences are detailed in brackets. Indeterminate patterns for G3 are also described in brackets. PVLs are expressed as HTLV-1 copies/100 PBMCs.

Sample N° (Sequence Code)	Group	Age	Gender	PVL
1	1- Leukemia	53	M	33.9768
2	1- Leukemia	66	F	40.1995
3 (ATL1)	1- Lymphoma	48	F	1.2974
4 (ATL2)	1- Leukemia	67	F	12.4920
5	1- HAM/TSP	43	F	0.7081
6	1- HAM/TSP	14	F	8.9051
7	1- HAM/TSP	27	F	3.1244
8	1- HAM/TSP	38	F	1.6633
9	1- HAM/TSP	52	F	8.5679
10	1- HAM/TSP	65	M	5.3882
11	1- HAM/TSP	37	F	1.2808
12	1- HAM/TSP	51	F	4.7829
13 (Neu28)	1- HAM/TSP	42	F	13.0850
14 (Neu14)	1- HAM/TSP	39	F	1.0119
15	1- HAM/TSP	52	M	35.0971
16	1- HAM/TSP	59	F	12.8610
17	1- HAM/TSP	50	M	1.3508
18	1- HAM/TSP	56	F	29.5394
19	1- HAM/TSP	NA	M	0.1183
20	1- HAM/TSP	26	F	3.1234
21	1- HAM/TSP	71	F	10.4542
22	1- HAM/TSP	35	M	0.5227
23	1- HAM/TSP	67	F	1.6661
24	1- HAM/TSP	52	M	15.5252
25	1- HAM/TSP	49	M	4.0326
26	2	50	M	12.4340
27	2	64	M	1.8929
28	2	50	M	0.0832
29	2	46	F	1.2681
30 (ASYAR3)	2	35	M	4.6143
31 (ASYAR2)	2	26	F	0.7502
32	2	47	F	0.2476
33	2	25	M	8.4668
34	2	33	M	2.3861
35	2	39	F	2.8778
36 (ASYAR1)	2	47	F	0.4813
37	2	52	F	<3 copies/ reaction
38	2	NA	F	<3 copies/ reaction
39	2	38	F	0.1832
40 (BDAR20)	2	38	M	3.9461
41	2	57	M	5.9663
42	2	NA	M	<3 copies/ reaction
43	2	46	M	10.5358
44	3 (p19)	59	M	0.0013
45	3 (p19)	24	M	0.3365
46 (BDAR21)	3 (GD21)	28	M	0.1493
47 (BDAR18)	3 (p24, GD21)	52	M	0.1452
48 (FSW8)	3 (HGIP)	32	F	<3 copies/ reaction
49 (FSW9)	3 (p19)	52	F	<3 copies/ reaction
50 (FSW7)	3 (p19)	25	F	<3 copies/ reaction
51 (BDAR19)	3 (GD21)	31	M	<3 copies/ reaction
52	3 (HGIP)	21	M	*pol* not detected

**Table 4:** Individual proviral load values (PVL) distribution by groups (G). G1: positive samples by Western blot (WB) from individuals with pathology; G2: positive samples by WB from asymptomatic carriers (AC); and G3: indeterminate samples by WB from AC. PVLs are expressed as HTLV-1 copies/100 PBMCs.

PVL Range	G1 (*n*=25)	G2 (*n*=18)	G3 (*n*=8)
<1	12%	44.4%	100%
1–10	52%	44.4%	-
>10	36%	11.2%	-

### 2.5. PVL Association with Gender, Age, and Optical Density

**Figure 3:**
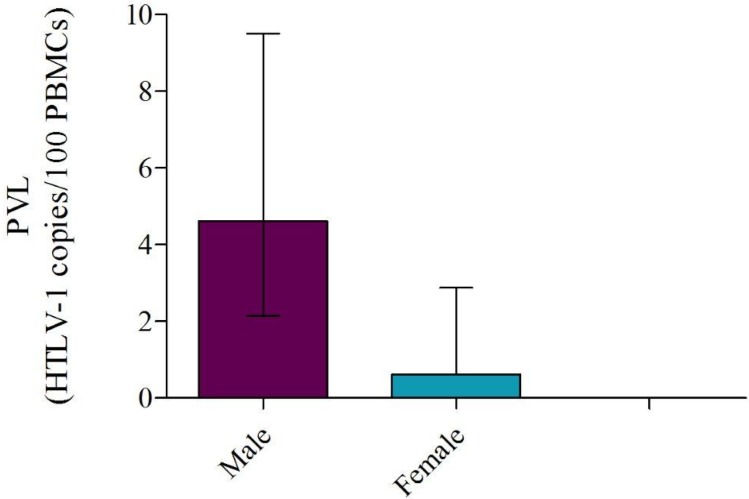
Median individual proviral loads (PVL) of G2 samples distrubuted by gender. A significant association (p=0.01) is observed between PVL and gender in Western blot (WB) positive samples from asymptomatic carriers (AC) distributed as: Males (*n* = 9) and Females (*n* = 6). Median PVL values were 4.61 in males and 0.61 in females. Median values together with interquartile ranges are shown.

A moderate correlation (S=0.56) between PVL values and age at the moment of the sample extraction was observed in G1, which included patients with pathology who were not in treatment. Meanwhile, there was a significant association between PVL and gender in G2 (*p* = 0.01) (Figure 3). No correlation was observed in G3. Significantly lower optical density values were observed in most of the seroindeterminatesamples (*n* = 6/7), when compared with plasma samples from G1 and G2 selected randomly.

### 2.6. HTLV-1 Phylogeny

A rooted neighbor-joining (NJ) tree of 134 HTLV-1 strains based upon a 458-bp fragment of the LTR region was performed, including 14 new strains from Argentina (G1: *n*=4; G2: *n* = 4; G3: *n* = 6); three G3 sequences could not be obtained. Cosmopolitan subtype HTLV-1a was clearly separated from HTLV-1 subtypes b, c, d, e, f, and g, with a bootstrap value of 69%. Within the cosmopolitan subtype, five subgroups were identified as previously described [4]. Although all of them were consistently separated by NJ, only two subgroups, West African/Caribbean subgroup C and North African subgroup D were well supported, showing higher bootstrap values than 75% (90% and 96%, respectively). Thirteen strains clustered within the cosmopolitan subtype transcontinental subgroup A, while the remaining one (N° 13, Neu28) from G1 clustered among sequences from Brazil and Peru previously reported by our group as Divergent Strains (Figure S2). Of these, five strains (ASYAR2, N°31; BDAR20, N°40; ASYAR3, N°30; ATL2, N°4; and Neu14, N°14) grouped with sequences from the Big Latin American cluster and three sequences (BDAR21, N°46; ATL1, N°3; and BDAR19, N°51) from the Small Latin American cluster. All eight sequences from the Latin American clusters grouped with Amerindian strains and other populations from Argentina previously reported by our group. One of the G3 strains (FSW7, N°50) grouped in the South African cluster along with one Argentine blood donor (BD1) and one Peruvian female sex worker (FSW1) residing in Argentina. The remaining four (BDAR18, N°47; FSW8, N°48; FSW9, N°49; and ASYAR1, N°36) clustered within the transcontinental group, near the other Argentine sequences previously reported by our group but not in any specific cluster.

### 2.7. Sequence Analysis

LTR and *tax* genes could be amplified in eight and four of all the seroindeterminate cases analyzed, respectively. Of those, six LTR and two *tax* sequences could be obtained with high quality, despite having repeated the procedure several times and assayed different DNA amounts with the remaining samples. A total of 25 mutations were detected among LTR sequences from G3 samples, of which seven were linked to geographical subtypes. Four mutations were observed in more than one sequence and the remaining 14 in only one, as detailed in Table 5. Of them, one mutation in the TRE-1 domain was present in the distal (dr) and one in the central (cr) region of the LTR. Regarding the TRE-2, two punctual mutations (8522T>C, 8545G>A) were observed in sample N° 50, while the other three were present in most Argentine sequences. While not found in any of the LTR sequences from G3, a mutation was observed in the distal region of TRE-1 (8403A>G) in 50% of the remaining sequences, which corresponded to samples with positive HTLV-1 results by WB and a significant difference (*p* = 0.03) between these frequencies was established. As for sequences from patients with pathology, eight (2 ATL and 6 HAM/TSP) out of 15 had this same mutation, thus maintaining the significance (*p* = 0.04).

*Tax* sequences showed 12 punctual mutations at the nucleotide level; of them, five were present in most of the Argentine sequences. Through the analysis of non-synonymous (NS) mutations in functional domains of the Tax protein, four amino acidic mutations were detected, two of them corresponding to geographical subtypes (A221V, S304N). The remaining mutations (H43P and M154V) were detected in sequences N° 46 and 47, respectively; H43P is located within the Nuclear Localization Signal domain, the Zn Finger, and CREB activation by Tax, while M154V is in the NF-ĸB activation region and the Dimerization Domain.

**Table 5:** Punctual mutations detected in LTR (U-3, R, and U-5) and *tax* gene sequences of indeterminate Western Blot samples confirmed as HTLV-1 positive by nested PCR. Six LTR and two *tax* sequences from seroindeterminate samples were analyzed, together with four sequences from HTLV-1 patients with pathology, four from HTLV-1 asymptomatic carriers, and 44 sequences from Argentina, previously obtained by our group and available in Gene Bank. Geographical: mutation linked to geographical subtypes; dr: distal region; cr: central region.

Punctual Mutation	Sequence N°	Region	Punctual Mutation	Sequence N°	Region
8295G>A	46- 48	LTR; U-3	8718C>T	51	LTR; R
8367C>A	Geographical	LTR; U-3	8779T>C	47	LTR; R
8381G>A	48	LTR; U-3	8822G>A	46	LTR; R
8391G>A	46	LTR; U-3	8828A>G	47	LTR; R
8392G>A	46	LTR; U-3	8912T>C	46- 47	LTR; U-5
8420C>T	49	LTR; U-3; TRE-1; dr	8955G>A	47	LTR; U-5
8428_8429insA	Geographical	LTR; U-3; TRE-1	7383C>T	46	*tax*
8446G>A	Geographical	LTR; U-3; TRE-1	7398C>T	46- 47	*tax*
8471G>T	50	LTR; U-3; TRE-1; cr	7401C>T	Geographical	*tax*
8509A>G	Geographical	LTR; U-3; TRE-2	7431G>A	47	*tax*
8509_8511delA	Geographical	LTR; U-3; TRE-2	7448A>C	46	*tax*
8522T>C	50	LTR; U-3; TRE-2	7780A>G	47	*tax*
8545G>A	50	LTR; U-3; TRE-2	7914T>C	Geographical	*tax*
8546T>C	Geographical	LTR; U-3; TRE-2	7920C>T	Geographical	*tax*
8606C>G	Geographical	LTR; U-3	7933C>T	47	*tax*
8606C>A	49- 51	LTR; U-3	7982C>T	Geographical	*tax*
8632G>A	46	LTR; U-3	8001A>G	46	*tax*
8655G>T	46	LTR; U-3	8231G>A	Geographical	*tax*
8665C>T	46- 48	LTR; R			

## 3. Materials and Methods

### 3.1. Samples

A retrospective cross-sectional study was carried out on four different populations. Of those, Injecting Drug Users (IDU; *n* = 173), Female Sex Workers (FSW; *n* = 613), and Men who Have Sex with Men (MSM; *n* = 667) have been previously recruited and studied by our group; the corresponding HTLV-1/2 prevalence was reported in Berini *et al*. [26]. The fourth population included individuals with previous serological reactive results for HTLV-1/2 and/or with symptoms of HTLV-1 associated pathologies, whose samples have been sent from blood banks and/or hospitals from all over Argentina to our Institute for HTLV diagnosis or confirmation (HDC) between January 2011 and January 2014. The prevalence of the former population is reported here. All participants provided a written informed consent and the project was approved by the Institutional Review Board of Nexo Civil Association, Argentina. Samples confirmed as HTLV-1 positive by molecular techniques were classified in three different groups (G). G1 consisted of all the positive samples by WB from individuals with pathology who were not on treatment received for HDC between 2011 and 2014: 21 HAM/TSP patients and 4 Acute Leukemia Lymphoma (ATLL) patients. G2 included 18 WB positive samples from asymptomatic carriers (AC), who were chosen randomly for this study. G3 included 9 indeterminate samples by WB, also from AC. Serological and socio-demographic information of all nine G3 individuals is described in Supplementary Table 1.

### 3.2. Diagnostic Algorithm

Antibody screening for HTLV-1/2 was performed by particle agglutination technique (SERODIA-HTLV-I, Fujirebio, Tokyo, Japan) and/or by enzyme-linked immunosorbent assay (ELISA) (Murex HTLV-I+II, Abbott Laboratories Argentina, Buenos Aires, Argentina or HTLV I&II Ab v. ULTRA, Dia.Pro, Milan, Italy). Reactive samples were subjected to WB confirmation (HTLV blot 2.4, Genelabs Diagnostics, Science Park, Singapore). A WB was scored as HTLV-1 or HTLV-2 positive, untypeable, indeterminate, or negative according to the manufacturer’s criteria.

For molecular confirmation of indeterminate or HTLV-positive samples by WB, DNA was extracted from peripheral blood mononuclear cells (PBMCs) by column extraction (ADN PuriPrep-S kit, Highway^®^, Inbio, Tandil, Argentina) and analyzed with ‘‘in-house’’ n-PCR for HTLV-1 and 2 *pol* and *tax* regions as previously described [19,20]. PCR was considered positive when amplicons from at least one amplification reaction were clearly detectable following agarose gel analysis [11]. Samples with non-reactive results by PA or ELISA were also further confirmed by n-PCR in order to avoid misdiagnosis in patients on retroviral treatment (because of other infections) and immune-compromised patients.

### 3.3. DNA Quantitation

Absolute quantitation of PVL was performed by real-time SYBR Green PCR, using an ABI Prism 7500Prism System (Applied Biosystems, Foster City, CA, USA).The HTLV-1 *pol* gene was amplified using 5 µL DNA, 12.5 µL SYBR Green PCR Master Mix (Applied Biosystems), and 200 nM of each primer (SK110-1: 5’-CCCTACAATCCAACCAGCTCAG-3’ and SK111-1: 5’-GTGGTGAAGCTGCCATCGGGTTTT-3’). PCR amplification of the albumin gene (ALB-S: 5’-GCTGTCATCTCTTGTGGGCTGT-3’ and ALB-AS: 5’-AAACTCATGGG AGCTGCTGGTT-3’) was performed as a separate reaction, as an endogenous reference to avoid variation due to differences in either the PBMC number or the DNA extraction method used. Cycle conditions were the following: 2 min at 50 °C and 10 min at 95 °C followed by 40 cycles of 15 s at 95 °C and 1 min at 65 °C. Melting curves were performed after the end of the amplification cycles to validate the specificity of the amplified products. Standard curves were generated using 10-fold serial dilutions of DNA from MT2 cells (10^4^–10^0^), and normalized to two copies of the HTLV-1 *pol* gene and two copies of the cellular albumin gene per MT2 cell [27]. All standard dilutions, controls, and individual samples were run in triplicate for both HTLV-1 and albumin DNA quantitation. Standard curves were accepted when slopes were between –3.10 and –3.74 and the R^2^ was >0.99 [28]. The accuracy of the diagnostic test was assessed by measuring intra-assay and inter-assay variability. Intra-assay variability was evaluated by calculating the coefficient of variation (CV) of three viral load replicates from three DNA samples in three different ranges defined as low (<1 HTLV-1 copy/100 cells), medium (1–10 HTLV-1 copies/100 cells), and high (>10 HTLV-1 copies/100 cells). Inter-assay variability was calculated by measuring the CV for a high PVL sample in three independent runs. CV rather than standard deviations were used as they are not affected by the PVL absolute value. HTLV-1 proviral load was reported as [(*pol* average copy number)/(albumin average copy number/2)]*100 and expressed as the number of HTLV-1 copies/100 cells.

### 3.4. Molecular Analysis

Indeterminate samples were subjected to hemi-nPCR, aimed at amplifying LTR and *tax* genes. Amplification of the 3’ LTR region was performed using 8200LA (5’-CTCACACGGCCTCATACAGTACTC -3’) and R2 (5’-GTGCTATAGGATGGGCTGTCGC-3’) as outer primers and 3VINT (5’-GAACGCRACTCAACCGGCRYGGATGG-3’) and 3LTRf (5’-TCCCCATTTCTCTATTTTTAACG-3’) as inner primers (528 bp, ATK-1 genome position 8196–8699). Amplification of the *tax* region was carried out with outer primers HFL75 (5’-GCTATAGTCTCCTCCCCCTGC-3’) and 3VINT (5’-GAACGCRACTCAACCGGCRYGGATGG-3’) and inner primers TaxF (5’-ATGGCCCACTTCCCAGGGTT-3’) and TaxR (5’-TCAGACTTCTGTTTCTCGGA-3’), specific for HTLV-1. A slight modification was made to the protocols described elsewhere, in order to amplify *tax* and LTR genes in seroindeterminate samples by enhancing the DNA amount [29]. Direct sequencing reactions were done using a Big Dye Terminator 3.1 Cycle Sequencing RR-100 (Applied Biosystems). Sequences were generated on a 3500xL Genetic Analyzer AB/HITACHI according to the manufacturer’s instructions. Sequences were edited manually (Sequencher 4.8) and then aligned using Clustal W (BioEdit 7.0.4.1 sequence alignment editor). For the sequence analysis of both genes, the ATK-1 genome was included as a reference prototype sequence and four samples from G1 together with four samples from G2 included as controls. Furthermore, 50 LTR sequences were also added, all of them corresponding to samples from Argentina and available in PubMed. MEGA software v. 5.2.2 was used for translation of *tax* gene sequences, based on the conventional genetic code.

To construct a comprehensive phylogenetic dataset, 14 of the LTR sequences (six seroindeterminate samples, four from HTLV-1 patients with pathology, and four from HTLV-1 AC) (See 5.5 “Accession numbers”) were aligned along with 120 HTLV-1 reference strains obtained from the GenBank database, preferentially chosen because they were either from Argentina or from neighboring countries with high migration rates to Argentina. The Mel 5 reference strain (Melanesian origin, subtype c) was used as an outgroup. Once aligned, the dataset consisted of 458 bp corresponding to the 3´ LTR region. The phylogenetic analysis was performed by neighbor joining (NJ) using MEGA 5.2 and the tree topology was visualized with TreeView (http://taxonomy.zoology.gla.ac.uk/rod/treeview.html) [30].

### 3.5. Accession Numbers

Accession numbers corresponding to all new sequences mentioned here are detailed below. ASYAR2LTR: KT633516; BDAR20LTR: KT633517; FSW7LTR: KT633518; ASYAR3LTR: KT633519; BDAR19LTR: KT633520; FSW8LTR: KT633521; Neu14LTR: KT633522; BDAR18LTR: KT633523; BDAR21LTR: KT633524; FSW9LTR: KT633525; ASYAR1LTR: KT633526; ATL1tax: KT633527; Neu2tax: KT633528; ASYAR2tax: KT633529; BDAR18tax: KT633530; ATL2tax: KT633531; ASYAR1tax: KT633532; ASYAR3tax: KT633533; BDAR20tax: KT633534; Neu14tax: KT633535; BDAR21tax: KT633536. The following numbers correspond to the sequences obtained from GenBank for the molecular analysis. MEL5 (L02534); Efe1 (Y17014), ITIS (Z32527), PH236 (L76307); 2810YI (AY818432); Lib2 (Y17017); pyg19 (L76310); HS35 (DI3784), FrGu1 (AY324785), BO (U12804), Pr52 (U12806), Pr144 (U12807), Bl1.Peru (Y16481), RKl4.Peru (AF054627), BCl2.1 (U32557), H5 (M37299), Ni1-3.Peru (Y16484, Y16487, Y16485), ATL-YS (U19949), ATK-1 (J02029),MT4.LB ( Z31661), Br4 (AY324788), Bl3.Peru (Y16483), Neu13 (EU622623), Neu10 (EU622620), MT2 (L03562), 73RM (M81248), Ar11 (AY324777), FSW6 (EU622605), MSM2 (EU622609), BD7 (EU622588), Sur229-30 (AY374468, AY374466), BD3 (EU622586), BD10 (EU622590), TBH1-3 (L76026, L76025, L76034), FSW1 (EU622600), BD1 (EU622584), BD8 (EU622589), Gya468 (AY374459), Gya813 (AY374462), SurHM22 (AY374467), Gya542 (AY374460), BOI (L36905), BD13 (EU622593), BRRJ136.96 (DQ323759), KUW1-2 (L42253, L42255), IRN2 (U87261), CH26 (D23690), Abl.A (U87264), BCl1.2 (U32552), BRRP445 (DQ323755), BRRJ276.95 (DQ323750), BRRJ56.00 (DQ323754), BRRJ53.97 (DQ323753), Neu4 (EU622615), PW2 (EU622625), Neu5 (EU622616), MSM4 (EU622611), IDU4 (EU622608), Neu3 (EU622614), BD2 (EU622585), CAM (AF063819), BRRJMDP (DQ323751), ARGSOT (AF007755), AMA (X88871), CMC (X88872), TBH4 (L76028), BRRP495(DQ323755), FSW4 (EU622603), Me3.Peru (Y16480), Ar55 (AY324782), FSW5 (EU622604), Sur1597 (AY374465), Gya572 (AY374461), Neu1 (EU622612), FSW2 (EU622601), JCP (X88875), BRRJFA (DQ323757), Me1.Peru (Y16478), MASU (X88877), FCR (X88873), BRRJ86.97 (DQ323760), MAQS (X88876), Ar5 (AY324783), Qu2.Peru (Y16476), Me2.Peru (Y16479), Qu3.Peru (Y16477), J37 (FJ751855), BD16 (EU622596), IDU1-3 (EU622598, EU622606, EU622607), BD15 (EU622595), BD14 (EU622594), BD12 (EU622592), Neu11 (EU622621), BD11 (EU622591), J77 (FJ758161), J43 (FJ751856), BD4 (EU622587), J68 (FJ751858), J20 (FJ751854), Neu12 (EU622622), Ar49 (AY324793), Ar15 (AY324778), PW1 (EU622624), FSW3 (EU622602), Qu1.Peru (Y16475), Neu7 (EU622618), ARGDOU (AF007751), Neu8 (EU622619), BD17 (EU622597), J47 (FJ751857), MSM3 (EU622610), Neu2 (EU622613).

### 3.6.Statistical Analysis

Data analysis was performed using the Kruskal–Wallis non-parametric method; when two groups were compared, the Mann–Whitney–Wilcoxon test was used. To evaluate the presence of association between PVL and age of individuals, correlation was determined based on Spearman coefficient (S). GraphPad Prism (version 6.03) software was applied and significant differences were defined as *p* < 0.05.

## 4. Discussion

HTLV indeterminate WB patterns have been reported worldwide, although they are more frequent in tropical areas [31,32]. Most of these reports refer to blood donors, and seroindeterminate frequency varies according to HTLV-1/2 endemicity (*i.e.*, the geographical area studied) [27,33]. In this study, we report for the first time in our country the prevalence of indeterminate WB results among at-risk populations: 2.31%, 2.12%, and 1.65% for IDUs, FSW, and MSM, respectively. Moreover, the prevalence of indeterminate WB patterns (5.19%) in the HDC population recruited in our Institute was higher than the one reported in our country for blood donors (0.1%), as expected due to a biased population [27]. On the other hand, it must be considered that screening in our laboratory has always been performed with the most efficient assays available in the country. Concerning the indeterminate WB banding patterns in the HDC population (both positive and negative cases by n-PCR), GD21 (alone or with other bands) and HGIP were the most frequent. Several studies have demonstrated the presence of HGIP patterns mostly among blood donors, and it has been suggested that generally these are not caused by HTLV-1 infection [32]. Nonetheless, as reported previously by our group, two HGIP cases corresponded to samples that turned out positive for HTLV-1 infection (one blood donor and one IDU) [10]. Regarding the “N pattern” recently described by Filippone *et al*., it was not observed neither in the HDC population, nor in at-risk populations (pattern described in Berini *et al* [26]) [11].

There are few reports suggesting that low HTLV-1/2 PVL could cause indeterminate WB patterns in samples from infected individuals [16,34]. Regarding HTLV-1, low PVL levels for these cases were suggested, especially in the ones in which no PVL could be successfully quantified, although no direct comparisons with seropositive patients were performed [35,36,37]. In this study, PVL values were compared between two groups with positive WB results: G1 (individuals with pathology without retroviral treatment) and G2 (asymptomatic carriers: AC). A significant difference was observed between them, in line with previously published data [21,22]. Furthermore, a third group was included consisting of nine samples from AC with seroindeterminate WB, of which four PVLs could be determined. Even when considering the small sample size of G3, a significant difference was observed between these three groups. Although a seroconversion could not be discarded in the indeterminate cases, these data demonstrate that in some cases indeterminate WB results could be associated with low HTLV-1 PVL; a low viral replication rate may consequently trigger a weak immune response and low concentrations of anti-HTLV-1 antibodies. 

Regarding age and gender, it was determined that these variables were associated with PVL levels among G1 and G2, respectively. While Vakili *et al*. observed no significant association between PVL in HAM/TSP patients and healthy carriers with age and gender, it has been reported that for both HTLV-1 and 2 infections, women have lower PVL levels than men, consistent with our results [38,39,40]. In contrast, Hisada *et al*. showed no gender differences in PVL [41]. Thus, further studies should be performed in order to clarify this issue. Regarding serological status, Manns *et al*. reported in 1999 that the anti-HTLV-1 antibody titer had a positive correlation with PVL levels, and years later, Akimoto *et al*. confirmed these results [42,43]. In this study, and similarly to what has been reported by Filippone *et al*., the optical density values obtained were lower for most of the seroindeterminate profile plasma samples when compared to HTLV-1 WB-positive ones [11].

As for phylogeny, all 14 new strains described in this study belonged to the cosmopolitan subtype and most of them classified within the transcontinental subgroup A (one sample classified as divergent). Half of the G3 sequences did not group in any specific cluster, while two grouped in the small Latin American cluster (BDAR19, BDAR21) and one in the South African cluster (FSW7), together with other sequences previously reported by our group (FSW1 and BD1) [44]. These data confirm our previous publication concerning the presence of HTLV-1 transcontinental and African strains circulating in Argentina, although most of the infected individuals in our study were not of black origin, supporting the hypothesis of multiple introductions of HTLV-1 of the cosmopolitan subtype in the New World [5].

Even though some of the observed mutations in LTR and *tax* genes were linked to geographical subtypes, others were further analyzed in order to establish their relevance, as it is well known that during replication and transcription the LTR/Tax system is extremely important. The promoter region LTR responds to the transactivation mediated by the Tax protein, which directly interacts by binding the DNA or indirectly by binding cellular transcription factors, in the Tax regulation elements known as TRE-1 and TRE-2 [45]. Although mutations in sequences from G3 were observed both in the distal and central regions of TRE-1, no significant associations were established given the number of mutated sequences. The same is valid for the TRE-2 region, which can also mediate transactivation by Tax-1 [46,47], considering that it contains binding sites for a large number of transcription factors, including AP-2, HNF-3, Ets family members, NFκB, and Sp1 [48]. Interestingly, we have not observed the 8403A>G mutation (distal region of TRE-1) in any of the G3 sequences, although it was present in 50% of the remaining sequences, all of them positive by WB. Therefore, a significant association was established between the presence of this mutation and seropositivity. Particularly, 53% of sequences from patients with pathology were mutated in base 8403, consistent with a report from Brazil, in which this mutation was significantly associated with high PVL values [17]. Regarding the Tax protein, non-synonymous mutations in the CREB activation domain as well as in the NF-ĸB and Zn Finger activation domains were detected. Previous data indicate that mutations in these regions displayed markedly attenuated abilities to transactivate the provirus and to reduce the ability to induce nuclear expression of NF-ĸB [18]. Whether the presence of mutations observed in this study could explain a diminished transactivation activity of Tax protein and therefore a low PVL still remains to be determined. Only functional studies would indicate their possible impact on indeterminate WB profiles.

Considering diagnosis, indeterminate WB results cannot be avoided without an improvement of serological commercial kits aimed at enhancing sensibility and specificity. Thus, and taking into account the high prevalence of these seroindeterminate cases worldwide [10,11,49], the usefulness of serological confirmation is questionable, highlighting the difficulties in interpretation and counseling. Seroindeterminate results represent a big challenge for health professionals, especially in those countries with endemic areas and no national programs for controlling HTLV infection. Furthermore, WB kits are far more expensive than n-PCR, being a relevant factor for the healthcare system. Costa and Thorstensson *et al.* have recommended different strategies for reducing costs and improving the accuracy of the diagnosis [24,50]. While both proposed two EIAs for screening, Costa recommends qPCR to confirm the infection. Even though qPCR is actually the standard method for PVL quantitation, other technologies have also been introduced. Recently, an mq-PCR testing algorithm for the diagnosis of HTLV-1/2 infection has been proposed [25]. Nevertheless, in some serologically confirmed positive cases, PVL could not be detected, especially in HTLV-2 samples [25,51]. In that context, Brunetto *et al*. reported the utility of digital droplet PCR (ddPCR) in the quantitation of HTLV-1 PVL [52]. They postulate that, even though both methods show a strong correlation and similar performance, ddPCR exhibits lower inter and intra-assay variability as it is based on a Poisson algorithm for quantitation of genes instead of the standard curve used in qPCR [52]. On the other hand, another study showed a higher sensitivity for qPCR compared to ddPCR when detecting cytomegalovirus in clinical samples [53]. Based on these data, more studies should be performed in order to establish the most efficient methodology for HTLV-1/2 PVL quantitation. Furthermore, a consensus regarding qPCR data interpretation and analysis for HTLV-1/2 PVL quantitation, as well as a universal expression unit, should be achieved in order to avoid confusion and misunderstanding. Besides, most HTLV-1/2 endemic areas correspond to developing countries and access to qPCR equipment is not always possible.

Therefore, we propose to re-evaluate the diagnostic algorithm, considering molecular confirmation by n-PCR for reactive samples, instead of qPCR, right after the combination of two screening tests. This alternative would avoid serological confirmation by WB, the most expensive stage of the diagnosis algorithm, until a better confirmation technique is available and standardized. Further studies with significant panels including HTLV-1, HTLV-2, and indeterminate samples should be carried out in order to establish whether it is time saving, effective, and less expensive.

## 5. Conclusions

This study describes the prevalence of indeterminate WB patterns among different populations from Argentina and demonstrates that in some cases these profiles may be associated with low HTLV-1 PVL. Mutations in LTR and *tax* have been described among both indeterminate and positive HTLV-1 cases, highlighting 8403A>G in the distal region of TRE-1, already related to high PVL. Still, the functional relevance of these mutations remains to be determined.

## Supplementary Files

Supplementary File 1

## References

[1] Gessain A., Cassar O. (2012). Epidemiological Aspects and World Distribution of HTLV-1 Infection. Front. Microbiol..

[2] Roucoux D.F., Murphy E.L. (2004). The epidemiology and disease outcomes of human T-lymphotropic virus type II. AIDS Rev..

[3] Gessain A., Mahieux R. (2000). Epidemiology, origin and genetic diversity of HTLV-1 retrovirus and STLV-1 simian affiliated retrovirus. Bull. Soc. Pathol. Exot..

[4] Vidal A.U., Gessain A., Yoshida M., Tekaia F., Garin B., Guillemain B., Schulz T., Farid R., Thé G. (1994). Phylogenetic classification of human T cell leukaemia/lymphoma virus type I genotypes in five major molecular and geographical subtypes. J. Gen. Virol..

[5] Van Dooren S., Gotuzzo E., Salemi M., Watts D., Audenaert E., Duwe S., Ellerbrok H., Grassmann R., Hagelberg E., Desmyter J. (1998). Evidence for a post-Columbian introduction of human T-cell lymphotropic virus in Latin America. J. Gen. Virol..

[6] Biglione M.M., Astarloa L., Salomón H.E. (2005). Referent HTLV-I/II Argentina Group. High prevalence of HTLV-I and HTLV-II among blood donors in Argentina: A South American health concern. AIDS Res. Hum. Retrovir..

[7] Gastaldello R., Hall W.W., Gallego S. (2004). Seroepidemiology of HTLV-I/II in Argentina: An overview. J. Acquir. Immune Defic. Syndr..

[8] (1988). Licensure of Screening Tests for Antibody to Human T-Lymphotropic Virus Type 1. MMWR.

[9] Khabbaz R.F., Heneine W., Grindon A., Hartley T.M., Shulman G., Kaplan J. (1992). Indeterminate HTLV serologic results in U.S. blood donors: Are they due to HTLV-I or HTLV-II?. J. Acquir. Immune Defic. Syndr..

[10] Berini C.A., Eirin M.E., Pando M.A., Biglione M.M. (2007). Human T-cell lymphotropic virus types I and II (HTLV-I and -II) infection among seroindeterminate cases in Argentina. J. Med. Virol..

[11] Filippone C., Bassot S., Betsem E., Tortevoye P., Guillotte M., Mercereau-Puijalon O., Plancoulaine S., Calattini S., Gessain A. (2012). A new and frequent human T-cell leukemia virus indeterminate Western blot pattern: Epidemiological determinants and PCR results in central African inhabitants. J. Clin. Microbiol..

[12] Prince H.E., Gross M. (2001). Impact of initial screening for human T-cell lymphotropic virus (HTLV) antibodies on efficiency of HTLV Western blotting. Clin. Diagn. Lab. Immunol..

[13] Mahieux R., Horal P., Mauclère P., Mercereau-Puijalon O., Guillotte M., Meertens L., Murphy E., Gessain A. (2000). Human T-cell lymphotropic virus type 1 gag indeterminate western blot patterns in Central Africa: Relationship to Plasmodium falciparum infection. J. Clin. Microbiol..

[14] Porter K.R., Liang L., Long J.W., Bangs M.J., Anthony R., Andersen E.M., Hayes C.G. (1994). Evidence for anti-Plasmodium falciparumantibodies that cross-react with human T-lymphotropic virus type I proteins in a population in Irian Jaya, Indonesia. Clin. Diagn. Lab Immunol..

[15] Hayes C.G., Burans J.P., Oberst R.B. (1991). Antibodies to human T lymphotropic virus type I in a population from the Philippines: Evidence for cross-reactivity with Plasmodium falciparum. J. Infect. Dis..

[16] Abrams A., Akahata Y., Jacobson S. (2011). The prevalence and significance of HTLV-I/II seroindeterminate Western blot patterns. Viruses.

[17] Netto E.C., Brites C. (2011). Characteristics of Chronic Pain and Its Impact on Quality of Life of Patients with HTLV-1-associated Myelopathy/Tropical Spastic Paraparesis (HAM/TSP). Clin. J. Pain..

[18] Smith M.R., Smith M.R. (1990). Identification of HTLV-I tax trans-activator mutants exhibiting novel transcriptional phenotypes. Genes Dev..

[19] Heneine W., Khabbaz R.F., Lal R.B., Kaplan J.E. (1992). Sensitive and specific polymerase chain reaction assays for diagnosis of human T-cell lymphotropic virus type I (HTLV-I) and HTLV-II infections in HTLV-I/II-seropositive individuals. J. Clin. Microbiol..

[20] Tuke P.W., Luton P., Garson J.A. (1992). Differential diagnosis of HTLV-I and HTLV-II infections by restriction enzyme analysis of 'nested' PCR products. J. Virol. Methods.

[21] Furtado M., Andrade R.G., Romanelli L.C., Ribeiro M.A., Ribas J.G., Torres E.B., Barbosa-Stancioli E.F., Proietti A.B., Martins M.L. (2012). Monitoring the HTLV-1 proviral load in the peripheral blood of asymptomatic carriers and patients with HTLV-associated myelopathy/tropical spastic paraparesis from a Brazilian cohort: ROC curve analysis to establish the threshold for risk disease. J. Med. Virol..

[22] Nagai M., Usuku K., Matsumoto W., Kodama D., Takenouchi N., Moritoyo T., Hashiguchi S., Ichinose M., Bangham C.R., Izumo S. (1998). Analysis of HTLV-I proviral load in 202 HAM/TSP patients and 243 asymptomatic HTLV-I carriers: High proviral load strongly predisposes to HAM/TSP. J. Neurovirol..

[23] Olindo S., Lézin A., Cabre P., Merle H., Saint-Vil M., Edimonana-Kaptue M., Signate A., Césaire R., Smadja D. (2005). HTLV-1 proviral load in peripheral blood mononuclear cells quantified in 100 HAM/TSP patients: A marker of disease progression. J. Neurol Sci..

[24] Costa E.A., Magri M.C., Caterino-de-Araujo A. (2011). The best algorithm to confirm the diagnosis of HTLV-1 and HTLV-2 in at-risk individuals from São Paulo, Brazil. J. Virol. Methods.

[25] Waters A., Oliveira A.L., Coughlan S., de Venecia C., Schor D., Leite A.C., Araújo A.Q., Hall W.W. (2011). Multiplex real-time PCR for the detection and quantitation of HTLV-1 and HTLV-2 proviral load: Addressing the issue of indeterminate HTLV results. J. Clin. Virol..

[26] Berini C.A., Pando M.A., Bautista C.T., Eirin M.E., Martinez-Peralta L., Weissenbacher M., Avila M.M., Biglione M.M. (2007). HTLV-1/2 among high-risk groups in Argentina: Molecular diagnosis and prevalence of different sexual transmitted infections. J. Med.Virol..

[27] Mangano A.M., Remesar M., del Pozo A., Sen L. (2004). Human T lymphotropic virus types I and II proviral sequences in Argentinian blood donors with indeterminate Western blot patterns. J. Med. Virol..

[28] Bustin S.A., Benes V., Garson J.A., Hellemans J., Huggett J., Kubista M., Mueller R., Nolan T., Pfaffl M.W., Shipley G.L. (2009). The MIQE guidelines: Minimum information for publication of quantitative real-time PCR experiments. Clin. Chem..

[29] Eirin M.E. (2011). Epidemiología molecular del virus linfotrópico T-humano tipo 1 (HTLV-1) en Argentina: Análisis étnico-geográfico y variabilidad viral. Doctoral Thesis.

[30] Gascuel O. (1997). BIONJ: An improved version of the NJ algorithm based on a simple model of sequence data. Mol. Biol. Evol..

[31] Cesaire R., Bera O., Maier H., Martial J., Ouka M., Kerob-Bauchet B., Ould Amar A.K., Vernant J.C. (1999). Seroindeterminate patterns and seroconversions to human T-lymphotropic virus type I positivity in blood donors from Martinique, French West Indies. Transfusion.

[32] Rouet F., Meertens L., Courouble G., Herrmann-Storck C., Pabingui R., Chancerel B., Abid A., Strobel M., Mauclere P., Gessain A. (2001). Serological, epidemiological, and molecular differences between human T-cell lymphotropic virus Type 1 (HTLV-1)-seropositive healthy carriers and persons with HTLV-I Gag indeterminate Western blot patterns from the Caribbean. J. Clin. Microbiol..

[33] (1996). The HTLV European Research Network. Seroepidemiology of the human T-cell leukaemia/lymphoma viruses in Europe. J. Acquir. Immune Defic. Syndr. Hum. Retrovirol..

[34] Olah I., Fukumori L.M., Smid J., de Oliveira A.C., Duarte A.J., Casseb J. (2010). Neither molecular diversity of the envelope, immunosuppression status, nor proviral load causes indeterminate HTLV western blot profiles in samples from human T-cell lymphotropic virus type 2 (HTLV-2)-infected individuals. J. Med. Virol..

[35] Yao K., Hisada M., Maloney E., Yoshihisa Y., Hanchard B., Wilks R., Rios M., Jacobson S. (2006). Human T Lymphotropic Virus Types I and II Western Blot Seroindeterminate Status and Its Association with Exposure to Prototype HTLV-I. J. Infect. Dis..

[36] Mangano A., Altamirano N., Remesar M., Bouzas M.B., Aulicino P., Zapiola I., DelPozo A., Sen L. (2011). HTLV-I proviral load in Argentinean subjects with indeterminate western blot patterns. Retrovirology.

[37] Demontis M.A., Hilburn S., Taylor G.P. (2013). Human T cell lymphotropic virus type 1 viral load variability and long-term trends in asymptomatic carriers and in patients with human T cell lymphotropic virus type 1-related diseases. AIDS Res. Hum. Retrovir..

[38] Vakili R., Sabet F., Aahmadi S., Boostani R., Rafatpanah H., Shamsian A., Rahim Rezaee S.A. (2013). Human T-lymphotropic Virus Type I (HTLV-I) Proviral Load and Clinical Features in Iranian HAM/TSP Patients: Comparison of HTLV-I Proviral Load in HAM/TSP Patients. Iran J. Basic Med. Sci..

[39] Hodson A., Laydon D., Bain B.J., Fields P.A., Taylor G.P. (2013). Pre-morbid human T-lymphotropicvirus type I proviral load, rather than percentage of abnormal lymphocytes, is associated with an increased risk of aggressive adult T-cell leukemia/lymphoma. Haematologica.

[40] Montanheiro P., Olah I., Fukumori L.M.I., Smid J., Penalva de Oliveira A.C., Kanzaki L.I.B., Fonseca L.A., Duarte A.J., Casseb J. (2008). Low DNA HTLV-2 proviral load among women in Sao Paulo City. Virus Res..

[41] Hisada M., Miley W.J., Biggar R.J. (2005). Provirus load is lower in human T lymphotropic virus (HTLV)-II carriers than in HTLV-I carriers: A key difference in viral pathogenesis?. J. Infect. Dis..

[42] Manns A., Miley W.J., Wilks R.J., Morgan O., Hanchard B., Wharfe G., Cranston B., Maloney E., Welles S., Blattner W.A. (1999). Quantitative Proviral DNA and Antibody Levels in the Natural History of HTLV-I Infection. J. Infect. Dis..

[43] Akimoto M., Kozako T., Sawada T., Matsushita K., Ozaki A., Hamada H., Kawada H., Yoshimitsu M., Tokunaga M., Haraguchi K. (2007). Anti-HTLV-1 tax antibody and tax-specific cytotoxic T lymphocyte are associated with a reduction in HTLV-1 proviral load in asymptomatic carriers. J. Med.Virol..

[44] Eirin M.E., Dilernia D.A., Berini C.A., Jones L.R., Pando M.A., Biglione M.M. (2008). Divergent strains of human T-lymphotropic virus type 1 (HTLV-1) within the Cosmopolitan subtype in Argentina. AIDS Res. Hum.Retrovir..

[45] Bosselut R., Lim F., Romond P.C., Frampton J., Brady J., Ghysdael J. (1992). Myb protein binds to multiple sites in the human T cell lymphotropic virus type 1 long terminal repeat and transactivates LTR-mediated expression. Virology.

[46] Marriott S.J., Boros I., Duvall J.F., Brady J.N. (1989). Indirect binding of human T-cell leukemia virus type I tax1 to a responsive element in the viral long terminal repeat. Mol. Cell. Biol..

[47] Numata N., Ohtani K., Niki M., Nakamura M., Sugamura K. (1991). Synergism between two distinct elements of the HTLV-I enhancer during activation by the trans-activator of HTLV-I. New Biol..

[48] Datta S., Kothari N. H., Fan H. (2000). *In vivo* genomic footprinting of the human T-cell leukemia virus type 1 (HTLV-1) long terminal repeat enhancer sequences in HTLV-1-infected human T-cell lines with different levels of Tax I activity. J. Virol..

[49] Costa J.M., Segurado A.C. (2009). Molecular evidence of human T-cell lymphotropic virus types 1 and 2 (HTLV-1 and HTLV-2) infections in HTLV seroindeterminate individuals from São Paulo, Brazil. J. Clin. Virol..

[50] Thorstensson R., Albert J., Andersson S. (2002). Strategies for diagnosis of HTLV-I and –II. Transfusion.

[51] Busch M.P., Switzer W.M., Murphy E.L., Thomson R., Heneine W. (2000). Absence of evidence of infection with divergent primate T-lymphotropic viruses in United States blood donors who have seroindeterminate HTLV test results. Transfusion.

[52] Brunetto G, Massoud G., Leibovitch E.C., Caruso B., Johnson K., Ohayon J., Fenton K., Cortese I., Jacobson S. (2014). Digital droplet PCR (ddPCR) for the precise quantification of human T-lymphotropic virus 1 proviral loads in peripheral blood and cerebrospinal fluid of HAM/TSP patients and identification of viral mutations. J. Neurovirol..

[53] Hayden R.T., Gu Z., Abdul-Ali D., Shi L., Pounds S., Caliendo A.M. (2013). Comparison of droplet digital PCR to real-time PCR for quantitative detection of cytomegalovirus. J. Clin. Microbiol..

