# Supplementary material for: Low Proviral Load is Associated with Indeterminate Western Blot Patterns in Human T-Cell Lymphotropic Virus Type 1 Infected Individuals: Could Punctual Mutations be Related?
Source: Viruses. 2015 Oct 28;7(11):5643–58. doi: 10.3390/v7112897 (PMC4664970; doi:10.3390/v7112897)
Supplement: Supplementary File 1 [file viruses-07-02897-s001.pdf]

Supplementary Information

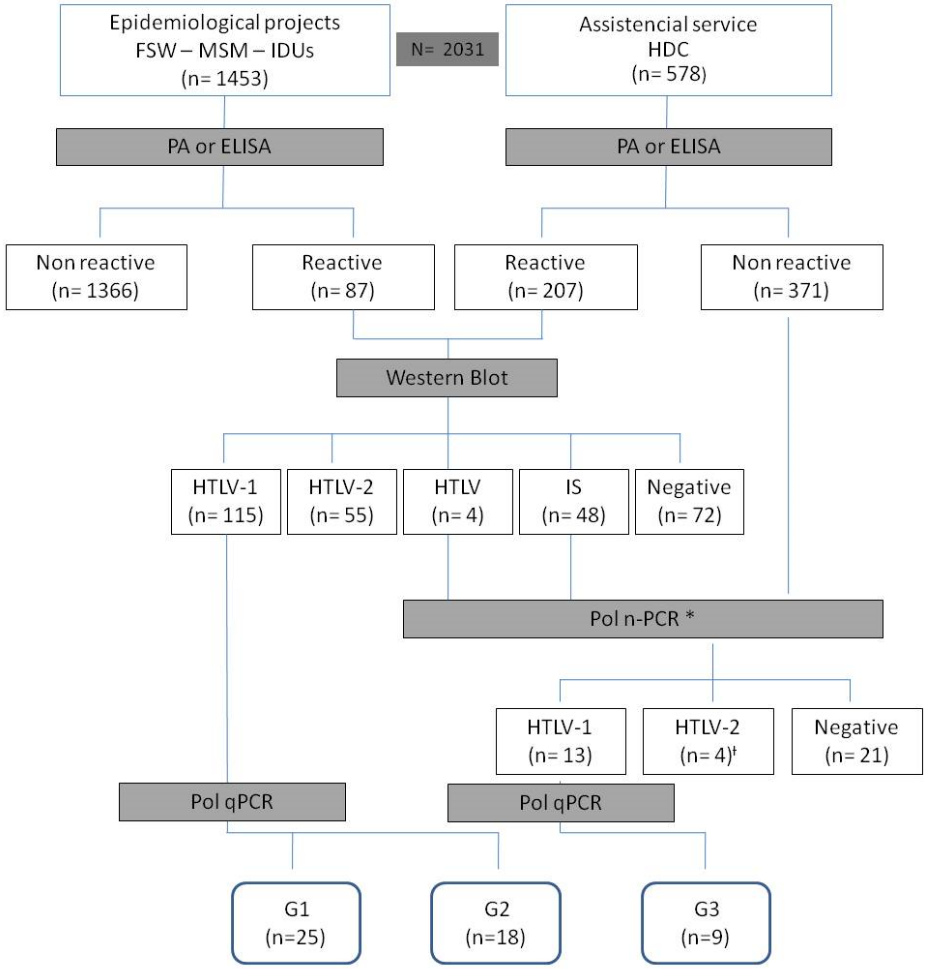

Figure S1. The global methodology is illustrated, including the number of samples tested at each step. Samples collected for epidemiological projects (MSM: Men who have Sex with Men; IDU: Injecting Drug Users; FSW: Female Sex Workers; *n* = 1453), as well as samples received thorough the assistance service (HDC: samples received at a Reference Institute for HTLV Diagnosis and Confirmation from blood banks or hospitals of Argentina; *n* = 578) were screened by particle agglutination (PA) or enzyme-linked immunosorbent assay (ELISA). Reactive samples from both studied populations (*n* = 294) were further tested by Western blot (WB): 115 and 87 samples were HTLV-1 and HTLV-2 positive, respectively. While 72 samples resulted negative by WB, four were not typeable HTLV and 48 were indeterminate samples (IS). Non-typeable HTLV samples, indeterminate ones, and non-reactive samples from the HDC population were tested by n-PCR. None of the latter tested positive. * Out of the 52 samples which needed molecular confirmation (48 IS+4 HTLV), 37 were tested by molecular techniques, , as no DNA was available for the others. ⁱ Two HTLV-2 samples were co-infected with HTLV-1 and HTLV-2, as reported by Berini *et al.* [1].

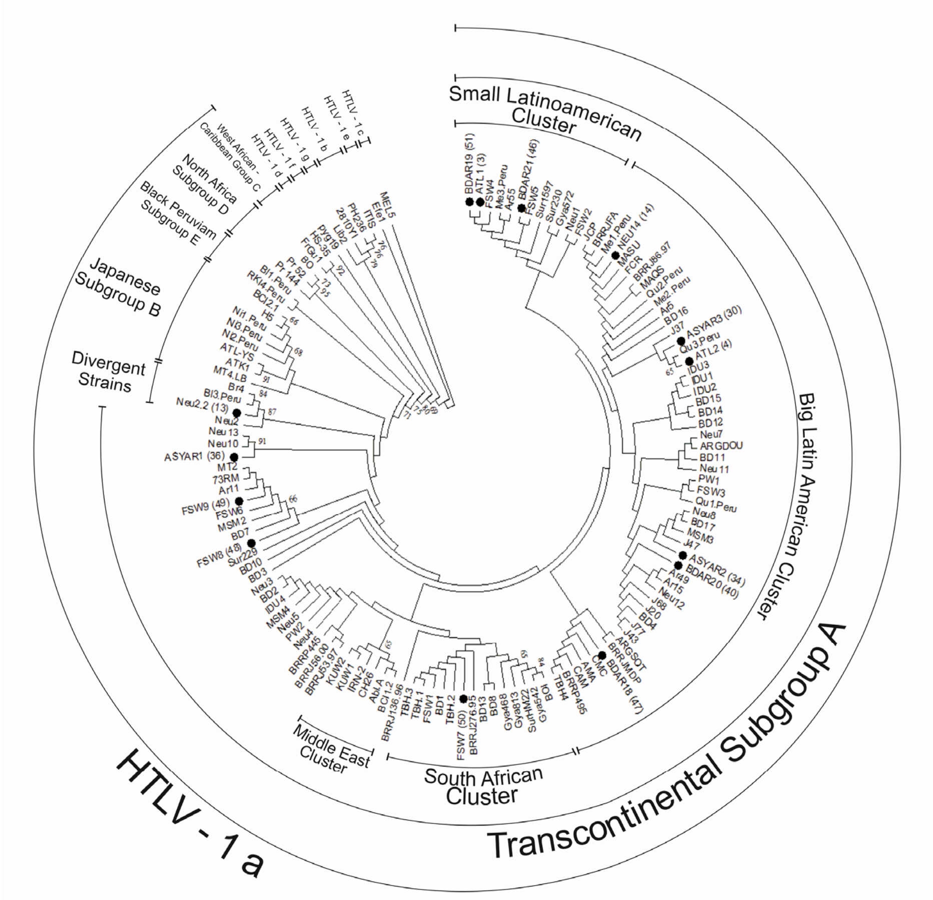

Figure S2. Rooted neighbor-joining tree of 134 HTLV-1 strains based upon a 458-bp fragment of the LTR region. All 14 Argentine new sequences are shown with a black dot (●). The HTLV-1 strain MEL5 was used as outgroup. Numbers on branches indicate the degree of support for each node. The geographic origin of reference strains included in this analysis is as follows: *HTLV-1c:* MEL5 (Solomon Islands); *HTLV-1e:* Efe1 (Zaire), *HTLV1b:* ITIS (Democratic Republic of Congo), PH236 (Gabon); *HTLV-1g:* 2810YI (Cameroon); *HTLV-1f:* Lib2 (Gabon); HTLV-1d: pyg19 (Central Africa Republic); *HTLV-1a:* HS35 (Caribbean), FrGu1 (French Guiana), BO (Algeria), Pr52 (Morocco), Pr144 (Morocco), Bl1.Peru (Peru), RKl4.Peru (Peru), BCl2.1 (British Colombia), H5 (Japan), Ni1-3.Peru (Peru), ATL-YS (United States, Japan), ATK-1 (Japan), MT4.LB (Japan), Br4 (Brazil), Bl3.Peru (Peru), Neu2.2 (Argentina), Neu2 (Argentina),Neu13 (Argentina), Neu10 (Argentina), ASYAR1 (Argentina), MT2 (Japan), 73RM (United States), Ar11 (Argentina), FSW9 (Argentina), FSW6 (Argentina), MSM2 (Argentina), BD7 (Argentina), FSW8 (Argentina), Sur229-30 (Suriname), BD10 (Argentina), BD3 (Argentina), Neu3 (Argentina), BD2 (Argentina), IDU4 (Argentina), MSM4 (Argentina), Neu5 (Argentina), PW2 (Argentina), Neu4 (Argentina), BRRP445 (Brazil), BRRJ56.00 (Brazil), BRRJ53.97 (Brazil), KUW1-2 (Kuwait), IRN2 (Iran), CH26 (Chile), Abl.A (South Africa), BCl1.2 (British Colombia), BRRJ136.96 (Brazil), TBH1-3 (South Africa), FSW1 (Argentina), BD1 (Argentina), FSW7 (Argentina), BRRJ276.95 (Brazil), BD13 (Argentina), BD8

(Argentina), Gya468 (Guyana), Gya813 (Guyana), SurHM22 (Suriname), Gya542 (Guyana), BOI (France), TBH4 (South Africa), BRRP495(Brazil), CAM (French Guiana), AMA (Brazil), CMC (Taiwan), BDAR18 (Argentina), BRRJMDP (Brazil), ARGSOT (Argentina), J43 (Argentina), J77 (Argentina), BD4 (Argentina), J20 (Argentina), J68 (Argentina), Neu12 (Argentina), Ar15 (Argentina), Ar49 (Argentina), BDAR20 (Argentina), ASYAR2 (Argentina), J47 (Argentina), MSM3 (Argentina), BD17 (Argentina), Neu8 (Argentina), Qu1.Peru (Peru), FSW3 (Argentina), PW1 (Argentina), Neu11 (Argentina), BD11 (Argentina), ARGDOU (Argentina), Neu7 (Argentina), BD12 (Argentina), BD14 (Argentina), BD15 (Argentina), IDU1-3 (Argentina), ATL2 (Argentina), Qu3.Peru (Peru), ASYAR3 (Argentina), J37 (Argentina), BD16 (Argentina), Ar5 (Argentina), Me2.Peru (Peru), Qu2.Peru (Peru), MAQS (Brazil), BRRJ86.97 (Brazil), FCR (Brazil), MASU (Brazil), Neu14 (Argentina), Me1.Peru (Peru), BRRJFA (Brazil), JCP (Brazil), FSW2 (Argentina), Neu1 (Argentina), Gya572 (Guyana), Sur230 (Suriname), Sur1597 (Suriname), FSW5 (Argentina), BDAR21 (Argentina), Ar55 (Argentina), Me3.Peru (Peru), FSW4 (Argentina), ATL1 (Argentina), BDAR19 (Argentina).

Table S1. Serological and socio-demographic information of seroindeterminate WB cases. G3 population (*n*=9) is described. MSM: Men who have Sex with Men; HDC: HTLV-1/2 Diagnosis and Confirmation; FSW: Female Sex Workers; IDU: Injecting Drug Users.

Sample N°	Population	Background information
44	MSM	Argentine. Negative for HIV, HBV, and HCV. No drug use. Post-secondary education
45	MSM	Argentine. Negative for HIV, HBV, and HCV. No drug use. Post-secondary education
46	HDC	Argentine. Blood donor from non-endemic area. Negative for HVB, HCV, HIV, and syphilis.
47	HDC	Argentine. Blood donor from non-endemic area. Negative for HVB, HCV, HIV, and syphilis.
48	HDC	Argentine. Blood donor from non-endemic area. Negative for HVB, HCV, HIV, and syphilis.
49	FSW	Argentine. Negative for HBV, HCV, HIV, and syphilis. No drug use. Primary education
50	FSW	Paraguayan. Negative for HBV, HCV, HIV, and syphilis. No drug use. Primary education
51	FSW	Argentine. Negative for HBV, HCV, HIV, and syphilis. No drug use. Primary education
52	UDI	Argentine. Negative for HIV. Positive for HCV and HBV. Primary education.

References

1. Berini, C.A.; Pando, M.A.; Bautista, C.T.; Eirin, M.E.; Martinez-Peralta, L.; Weissenbacher, M.; Avila, M.M.; Biglione, M.M. HTLV-1/2 among high-risk groups in Argentina: Molecular diagnosis and prevalence of different sexual transmitted infections. J. Med. Virol. 2007, 79, 1914–1920.
